# Outcomes of Surgical Site Infection Following Cranial Surgery From a UK Tertiary Center

**DOI:** 10.1093/ofid/ofag369

**Published:** 2026-06-20

**Authors:** Timothy P W Jones, Peter Davis, Oliver O’Sullivan, Mario Ganau, Jane Halliday, George Hadjipavlou, Matthew Scarborough, Monique I Andersson

**Affiliations:** Department of Infection, Oxford University Hospitals NHS Trust, Oxford, UK; Nuffield Department of Medicine, University of Oxford, Oxford, UK; Department of Infection, Oxford University Hospitals NHS Trust, Oxford, UK; Nuffield Department of Medicine, University of Oxford, Oxford, UK; Department of Infection, Oxford University Hospitals NHS Trust, Oxford, UK; Department of Neurosurgery, Oxford University Hospitals NHS Trust, Oxford, UK; Department of Neurosurgery, Manchester Centre for Clinical Neurosciences, Manchester, UK; Department of Anaesthetics, Oxford University Hospitals NHS Trust, Oxford, UK; Department of Infection, Oxford University Hospitals NHS Trust, Oxford, UK; Department of Infection, Oxford University Hospitals NHS Trust, Oxford, UK; Nuffield Division of Clinical Laboratory Science, Radcliffe Department of Medicine, University of Oxford, Oxford, UK

**Keywords:** central nervous system infections, neurosurgical procedures, postoperative complications, prostheses and implants, surgical wound infection

## Abstract

**Background:**

The evidence guiding management of cranial surgical site infections (SSIs) is sparse with limited data on optimal duration of antimicrobial therapy. There is a lack of published data on the approach to and outcomes of cranial SSI management.

**Methods:**

We retrospectively reviewed patients from Oxford University Hospitals, United Kingdom, requiring revision surgery for SSI following primary craniotomy, craniectomy, and cranioplasty between January 2019 and April 2022 and reviewed outcomes at 24 months. Data were collected on patient characteristics, diagnosis, management, and outcome. Analysis was performed in R.

**Results:**

Of 94 patients included, 44% were male and the median age was 51 years (IQR, 42–63). During surgical inspection, 40% of infections involved only superficial structures, 33% the extradural space, and 27% extended to the subdural space. *Staphylococcus aureus* (24%) and *Cutibacterium acnes* (22%) were the most frequently identified organisms, while all gram-negative bacilli combined accounted for 26% of cases. Rates of repeat surgery for infection were not significantly different between superficial and deep SSI (26% vs 28%, *P* = .499). On univariate analysis, corticosteroid use (odds ratio, 3.51; 95% CI, 1.24–10.00; *P* = .018) and prolonged antibiotic therapy (odds ratio, 1.34; 95% CI, 1.11–1.62; *P* = .002) were significantly associated with adverse outcome.

**Conclusions:**

These findings underscore the complexity of cranial SSIs and variation in presentation. Failure in this cohort was not uncommon, and practitioners should be particularly vigilant in the management of “superficial” infections. Developing a national consensus guideline would be an important step toward standardizing care and improving patient outcomes in this challenging cohort.

Advances in management of cranial pathologies and improved trauma care, coupled with an aging population, have meant a steady increase in the number of neurosurgical procedures that are performed in Europe and the United States [1, 2].

The incidence of surgical site infection (SSI) after cranial surgery has been reported as being between 5% and 33% [3, 4]. Cranial SSI carries a risk of mortality and morbidity from the effects of local infectious and inflammatory processes and indirectly as a result of the need for further neurosurgical procedures [5]. Despite this, there are no established national or international guidelines on the medical or surgical management of such infections.

Guideline development is challenging given the lack of robust clinical trial data and the absence of a specific classification for cranial SSIs, making comparison across studies difficult. Many of the neurosurgical treatment recommendations are extrapolated from device-related orthopaedic guidance, with little supportive neurosurgical data [6]. Adapted from prosthetic joint infection classification, some infections have been grouped temporally: “early” infections are defined as those occurring within 4 weeks of the procedure, “delayed” between 1 and 12 months of surgery, and “late” after 12 months [7]. Other classifications have focused on the depth of infection, adapting SSI guidelines from the Centers for Disease Control and Prevention [8, 9].

The general approach to management is ensuring adequate source control, with or without removal of prosthetic material, and prolonged courses of antimicrobial agents [6, 7]. The exact duration of antimicrobial therapy is poorly defined, with expert opinion recommending treatment durations from 4 to >12 weeks [7]. While some studies have highlighted risk factors, there is no published guidance on which specific factors might mandate prolonged courses of therapy. Although several studies have presented data on the incidence of cranial SSI after primary surgery, there are few cohorts detailing the outcome of cranial SSI management. A lack of consensus likely contributes to marked variation in practice. This may affect patient outcomes, cost to health care systems, and overuse of antibiotics, as shown in other infection syndromes [10].

We reviewed the surgical and antimicrobial management of cranial SSIs in a large tertiary service over a 5-year period to explore variation in practice and identify factors associated with poor outcome.

## METHODS

We retrospectively identified cases of cranial SSI requiring a surgical procedure from collated theater lists over a 40-month period (1 January 2019–27 April 2022) from a single large tertiary hospital. Emergency and elective operations were first screened to identify cases suggestive of infection related to all neurosurgery and were initially screened by the neurosurgery infection audit team. The clinical notes of the collated list of infection cases were reviewed again, and only cases of SSI involving craniotomy, craniectomy, and cranioplasty sites were included. Cases involving burr hole surgery were included where no prosthetic material had been inserted, and such cases were classified as craniotomies. SSI was defined as the presence of operative findings suggestive of infection, as discerned by the senior operating neurosurgeon (ie, pus, purulence, disrupted tissue layers, turbid fluid collection, or other findings) in line with definitions used in the national UK cranioplasty registry [11]. Cases that involved infections of other neurosurgical hardware, such as deep brain stimulators and external ventricular drains, were excluded. We excluded cases where the primary indication for initial neurosurgery was infective in origin (eg, brain abscess).

Data were extracted from electronic clinical records relating to patients' primary cranial neurosurgical procedure, infection surgery, and any subsequent neurosurgical interventions. Operative findings were identified via review of theater notes completed by the attending surgeon. Data were collected on the surgeon's opinion on the macroscopic depth of infection: *superficial infections*, superficial to the galea aponeurosis; *intermediate infections*, extending beyond the galea but not beneath the dura mater; *deep infections*, extending into the subdural space. The type of prosthetic material removed and remaining after operative management was recorded for each case.

The Laboratory Information Management System (Sunquest) was used to obtain microbiological data from theater specimens, including type of tissue cultured, the identified organisms, and antibiotic susceptibility. Operative samples in our institution were set up on a variety of selective agars appropriate to identify aerobic and anaerobic bacteria. This includes the use of fastidious anaerobic agar with neomycin and nalidixic acid tween agar, as well as the use of BD BACTEC lytic bottles. Cultures were continued for up to 10 days. Antibiotic regimens used pre- and postoperatively were collected from electronic prescriptions, including dose and route of administration. Clinical hematology and biochemistry results were obtained from the perioperative period and on completion of recommended antimicrobial therapies. These included preoperative and end-of-treatment C-reactive protein and white cell count and glycosylated hemoglobin. The presence of osteomyelitis was defined by histologic findings.

Patients were followed up for a minimum 2 years after infection surgery. Outcome data included duration of admission, mortality at 1 year, and need for further infection surgery at the site either prior to the cessation of antibiotic therapy or after completion of therapy. A composite primary endpoint was defined by treatment failure requiring repeat infection surgery within 2 years and all-cause mortality within 1 year of infection surgery.

### Statistical Analysis

Normally distributed continuous variables are presented as mean with standard deviation and were analyzed by *t* test with pairwise comparison as appropriate. Nonnormally distributed variables are reported as median and interquartile range and were analyzed by Wilcoxon rank sum and χ^2^ as appropriate.

Univariate and multivariate analyses of binary outcomes (ie, SSI treatment failure; repeat infection surgery and/or mortality) were performed with logistic regression. A threshold of *P*  *≤* .2 was used to define parameters for inclusion in multivariate analysis. All statistical analysis was conducted in R Statistical Software (version 4.4.1; R Core Team). This study is reported per the STROBE checklist for observational studies.

## RESULTS

**Figure 1.**
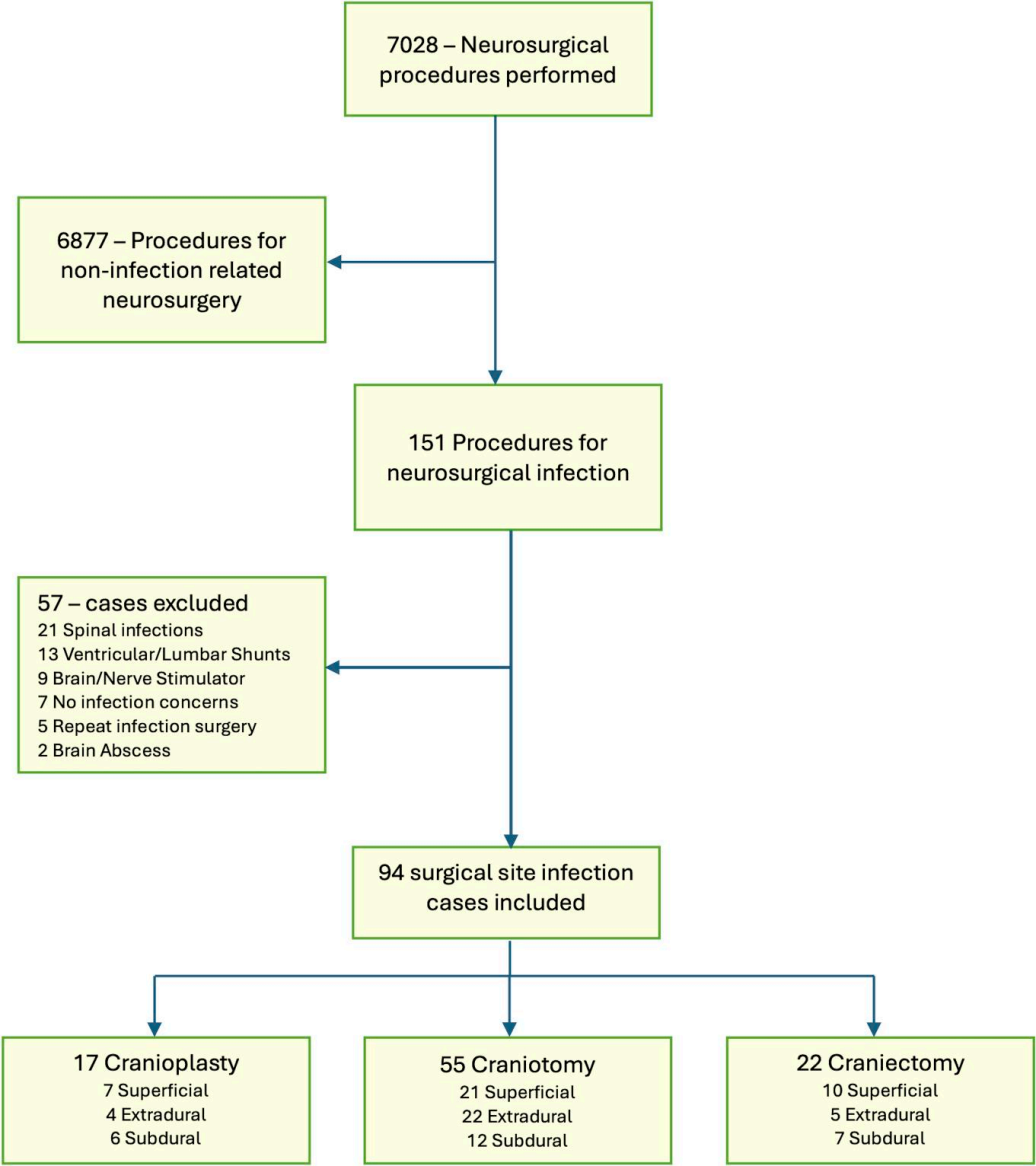
Schematic of cases included and excluded from initial screening.

During the study period, 7028 neurosurgical procedures were completed, of which 151 infection cases were captured by our initial screening and are reported in Figure 1. Of these, 94 unique patients with SSI following previous cranial surgery were identified. Baseline characteristics are shown in Table 1. The majority of infections were related to previous craniotomy surgery (n = 55, 59%), followed by craniectomy (n = 22, 23%) and previous cranioplasty surgery (n = 17, 18%).

**Table 1.** Baseline Characteristics and Operative Findings Separated by SSI and Intervention

	Type of Surgical Site Infection^a^			
	Total	Cranioplasty	Craniotomy	Craniectomy
Patients	94	17	55	22
Female	53 (56)	7 (42)	34 (62)	12 (55)
Age, y, mean (SD)	50.2 (14.7)	50.2 (14.2)	55.0 (13.9)	49.7 (16.7)
BMI, kg/m^2^, mean (SD)	28.0 (6.2)	26.9 (5.0)	29.0 (6.8)	26.5 (5.5)
Diabetes	12 (13)	2 (12)	8 (15)	2(9)
Corticosteroid use	19 (20)	4 (24)	13 (24)	2 (9)
Indication for primary neurosurgery				
Nonmalignant CNS tumor	39 (41)	7 (41)	27 (49)	5 (23)
Malignant primary CNS tumor	23 (24)	1 (6)	19 (35)	3 (14)
Metastasis	9 (10)	2 (12)	6 (11)	1 (5)
Bleed, stroke, trauma, other	23 (24)	7 (41)	3 (6)	13 (59)
Previous or current radiotherapy	18 (19)	3 (18)	14 (26)	1 (5)
Time between primary neurosurgical procedure and SSI, d, median (IQR)	34 (22–65)	36 (24–74)	36 (23–69)	25 (17–42)
Depth of infection				
Superficial	38 (40)	7 (41)	21 (38)	10 (46)
Extradural	29 (31)	4 (24)	21 (38)	4 (18)
Subdural	25 (27)	6 (35)	12 (22)	7 (32)
Osteomyelitis	1 (2)	…	1 (2)	1 (5)
Gross evidence of infection noted				
Yes	65 (69)	10	41	14
No	29 (31)	7	14	8
Management of flap/prosthesis				
Debridement only	26 (36)	5	21	…
Removal of bone flap/prosthesis	46 (64)	12	34	…
No. of samples sent for microbiology				
None	5 (5)	1	2	2
1	26 (28)	4	14	8
2	27 (29)	6	15	6
3	15 (16)	2	9	4
4	10 (11)	3	5	2
5	7 (7)	0	7	0
6	3 (3)	1	2	0
7	1 (1)	0	1	0

Abbreviations: BMI, body mass index; CNS, central nervous system; SSI, surgical site infection.

^a^Data are presented as No. (%) unless noted otherwise.

### Operative Findings

The largest subgroup (n = 38, 40%) of infections in the cohort was classed as superficial. Tissue samples were taken for microbiological analysis in 95% (89/94) of cases, although the number of samples varied (Table 1). Three or more samples were sent for microbiological analysis in 38% (36/94) of cases.

In the majority of cases, a decision was made to remove the infected prosthesis or bone flap (n = 46, 64%). Removal of prosthetic material was significantly more likely when the infection was deep (ie, extradural or subdural) than when there was infection of the skin and superficial soft tissues only (77% vs 18%, *P* < .001).

### Microbiological Findings

A total of 225 samples from 89 patients were processed by the microbiology laboratory. At least 1 organism was isolated in 71 patients (79.8%). Of infections where an organism was identified, 19% (n = 13) were polymicrobial in nature.

**Figure 2.**
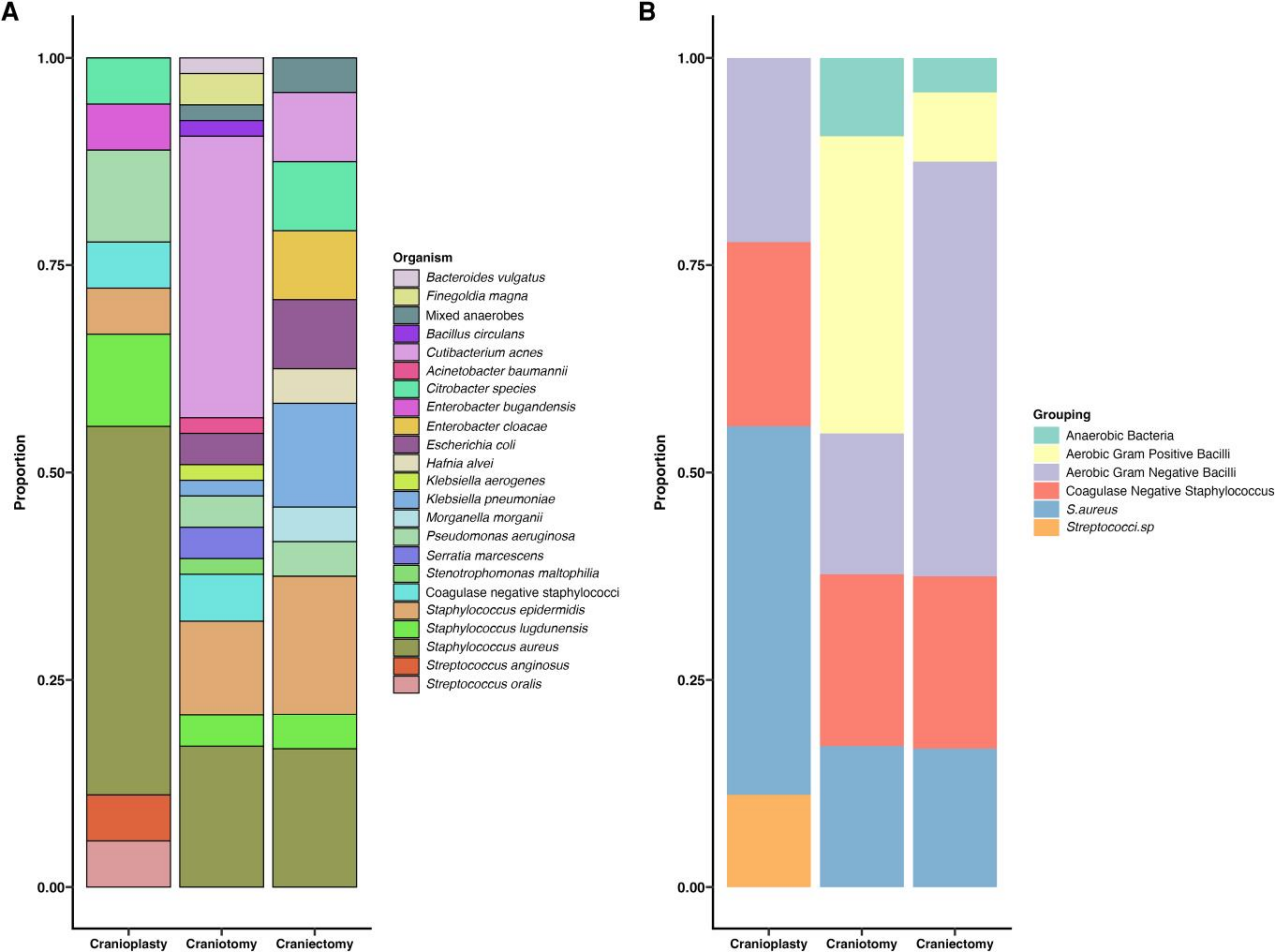
Plot of the relative frequency of organisms (*A*) and organism groups (*B*) separated by cranial surgical site infection identified in infection cases. Note that *Cutibacterium acnes* accounts for 20 of 21 cases in the gram-positive bacilli group.

*Staphylococcus aureus* (21/89, 24%) and *Cutibacterium acnes* (20/89, 22%) were the most frequently isolated organisms from surgical cases. Gram-negative bacilli (GNB) were found in samples from 26% (23/89) of cases; of these, *Pseudomonas aeruginosa* and *Escherichia coli* were most commonly identified (Figure 2*A*). Coagulase-negative staphylococci were isolated in 18% of cases (16/89), while anaerobes were less regularly identified (6%, 5/89). *S aureus* was significantly more likely to be present in cranioplasty SSI than in noncranioplasty SSI (odds ratio [OR], 6.50; 95% CI, 1.92–30.19; *P* = .004), while *C acnes* was more likely to be present in a craniotomy SSI than noncraniotomy infections (OR, 11.15; 95% CI, 2.83–74.78; *P* = .002; Supplementary Table 1). No samples cultured methicillin-resistant *S aureus*.

Given the large proportion of GNBs (Figure 2*B*), we performed a subgroup analysis. Patients with GNB had a significantly higher C-reactive protein than those without (median, 98.6 vs 12.7 mg/L; *P* = .002) and when compared with *S aureus* infections (median, 13.0 mg/L; *P* = .023). There was a trend for GNB infections to more commonly occur within 30 days of primary neurosurgery, although this did not reach significance (OR, 2.67; *P* = .081). GNBs were more likely to be seen in craniectomy SSI than noncraniectomy SSI (OR, 6.41; 95% CI, 2.03–22.26; *P* = .002). Although patients with GNB received a longer average duration of intravenous antibiotics (14 vs 7 days, *P* = .004), the median total antibiotic duration (intravenous and oral) was the same for each group (28 days). There was no association between GNB infections and an increased risk of treatment failure (*P* = .401).

### Antibiotic Management of SSI

Antibiotics were administered immediately prior to operative management and sampling in 44% (n = 41) of cases, with a median duration of 3 days (IQR, 1–5). The most frequent preoperative antibiotics administered were intravenous ceftazidime and intravenous vancomycin combination therapy (31%) or flucloxacillin monotherapy (26%). Administration of antibiotics prior to sampling was not associated with reduced odds of having positive microbiology in surgical samples (*P* = .561).

Ninety-three (99%) cases received intravenous postoperative antibiotics. The agents used are reported in Supplementary Table 2. Seventy-three (78%) patients switched to oral antibiotic therapy. The duration of antibiotic therapy was not significantly different among cranioplasty, craniotomy, and craniectomy SSIs (*P* = .743). Duration of therapy was significantly longer for deep infections that involved the subdural space (*P* = .002).

Complications related to antibiotic therapy occurred in 13 patients (14%). These were mostly mild, with rash occurring most frequently (n = 5). Risk of antibiotic complication was not associated with an increased antibiotic duration in this cohort (*P* = .899).

### Outcome of Treatment

The median length of stay was 10 days (IQR, 6–20), and individuals who had subdural infections had a longer length of stay than those with superficial infections (*P* < .001). Mortality at 1 year was 10% (9/94). Twenty-eight (30%) individuals met the composite endpoint for primary treatment failure (Table 2).

**Table 2.** Duration of Antibiotics and Outcome of Therapy Separated by Depth of Infection

	Depth of Infection^a^				
	Total (N = 94)	Soft Tissue (n = 38)	Extradural (n = 31)	Subdural (n = 25)	*P* Value
Postoperative antibiotic duration, d					
IV therapy	7 (4–14)	4 (4–7)	13 (4–14)	14 (9–28)	<.001
PO therapy	10 (4–28)	7 (4–14)	14 (0–21)	21 (0–28)	.581
Total therapy	24 (11–42)	14 (8–28)	21 (14–39)	42 (28–42)	.020
Length of stay, d	10 (6–20)	6 (4–11)	9 (6–16)	21 (10–27)	<.001
Outcomes					
Composite endpoint	28 (30)	11 (29)	8 (26)	9 (36)	.701
Further infection surgery	22 (23)	10 (26)	5 (16)	7 (28)	.499
Death at 1 y	9 (10)	1(3)	4(13)	4(16)	.156

Abbreviations: IV, intravenous; PO, oral.

^a^Data are presented as median (IQR) or No. (%).

**Figure 3.**
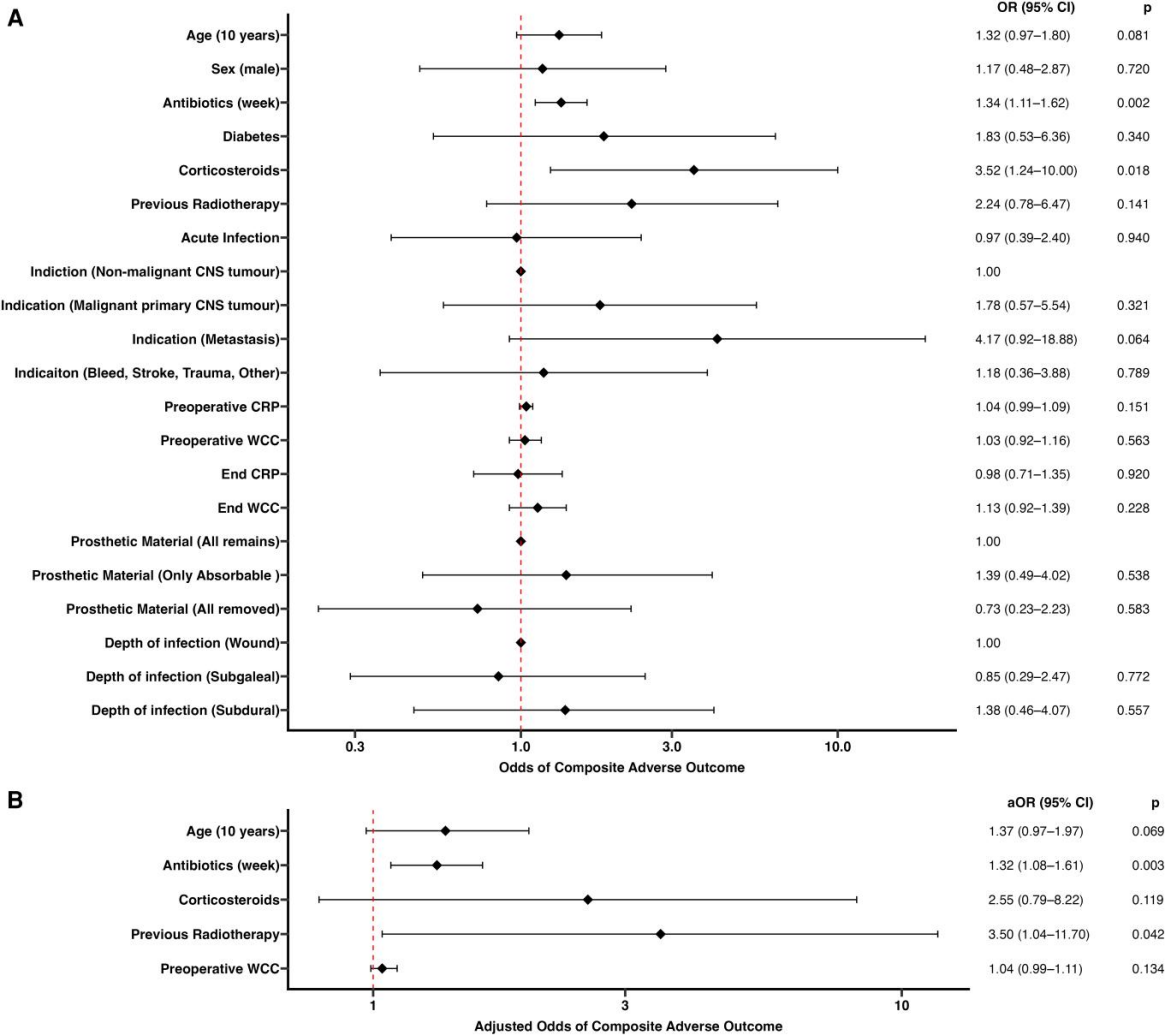
Forest plot of unadjusted (*A*) and adjusted (*B*) odds ratios of predictors of poor outcome after cranial surgical site infection. *P* values provided are for the likelihood ratio test other than multilevel components, where the Wald test *P* value is presented. For multilevel parameters, a likelihood test was used for consideration of significance in the multivariate model. aOR, adjusted odds ratio; CNS, central nervous system; CRP, C-reactive protein; OR, odds ratio; WCC, white cell count.

We assessed predictors of treatment failure. On univariate analysis, longer duration of antibiotic therapy (OR, 1.34; 95% CI, 1.11–1.62; *P* = .002) and current corticosteroid use (OR, 3.51; 95% CI, 1.24–10.00; *P* < .018) were significantly associated with adverse outcome (Figure 3*A*). In the multivariate model, duration of antibiotics(*P* = .003) and previous radiotherapy (*P* = .042) were associated with adverse outcome (Figure 3*B*).

## DISCUSSION

In this cohort, we found that treatment failure was common, with 30% of individuals meeting the composite endpoint for failure. This “failure” rate is similar to that reported in the other published cohorts [12, 13]. All-cause mortality rates associated with neurosurgical SSI were slightly lower in a national survey completed in 2017 in the United Kingdom [14]. However, this survey contained 65 cases of SSI, of which only 69% required an operative approach for management. Our cohort, which focused exclusively on outcomes of reoperations for SSI, is likely to represent more severe cases of SSI. Most cases of failure in this cohort were due to persistence of infection at the operative site and need for additional infection surgery. Similar failure rates have been reported in other single-center retrospective cohorts (32.2% and 26.1%) [12, 13].

Particularly notable was the finding that infections deemed to be superficial to the galea during surgery were no less likely to experience failure. Some experts suggest that distinguishing between deep and superficial infections in cranial SSIs is somewhat artificial, as there is a limited physical distance between superficial and deep tissues [6]. In our cohort, the most common reason for failure was the need for further infection surgery. As prosthetic material was retained in the majority of superficial infections (31/38 cases), some treatment failure could be mitigated by a lower threshold for removal of prosthetic material. There are limited data on the optimal surgical management of cranial SSIs. For deep SSIs, general consensus involves removal of flap or bone graft and delayed cranioplasty [15, 16]. While small cohorts have suggested that aggressive debridement can facilitate replacement of bone flaps or early replacement with a titanium mesh [16], further large studies are required to explore this approach. It is also important to highlight that removal of prosthetic materials and bone flaps is not without risk. Cranial bone deficits pose a risk of traumatic brain injury and an increased risk of neurologic deficits and hydrocephalus [17]. Debate remains over the optimum timing of cranioplasty and whether specific materials, such as porous hydroxyapatite, may have a role in reducing SSI rates [18, 19].

We also found that previous radiotherapy and concurrent corticosteroid use are both factors that increase the risk of SSI treatment failure. Predictors for treatment failure in cranial SSI are not well established, and there are no national or international guidelines on antibiotic duration or route of administration [7]. Corticosteroid use is well known to modulate immune function [20], and our findings support other studies that have shown that corticosteroid therapy increases the risk of other bacterial infections [21, 22]. There is limited research on the risk of corticosteroids on neurosurgical SSIs [23]. Local radiotherapy is also known to influence immune function in the short term [24], and it is plausible that damage to bone may underly the association that we found.

We also found an association between the duration of antibiotic therapy and risk of treatment failure. It is likely that physicians identified patients who were at greater risk of treatment failure and increased antibiotic durations accordingly. It is difficult to ascertain how these distinctions were made given the heterogenicity of the cases in this cohort and retrospective nature of the study. Indications for earlier surgical interventions, as well as approaches that better delineate only those patients who would benefit from prolonged courses of therapy, would be helpful to reduce the risk of antimicrobial resistance and toxicity [25].

Median antibiotic duration in our cohort was 24 days. Duration of therapy was longer in individuals with evidence of deep infection, which is in keeping with international practice for other soft tissue infections [26]. The majority of patients were treated at least in part with oral antibiotic therapy. Oral antibiotics have multiple benefits, including reduced cost to health care systems, shorter inpatient stay, and significant environmental benefits [27, 28]. Although their role in cranial SSI is not established, evidence can be inferred from orthopaedic and soft tissue infections [29]. The large proportion of individuals being prescribed oral antibiotics in this study and the lack of an association with treatment failure support this approach. The lack of national or international treatment recommendations hampers efforts to establish consistent management of cranial SSI, and the field would benefit from a consensus on treatment durations for common presentations. In light of this, we are currently establishing a national consortium to better delineate management strategies [30].

Although gram-positive organisms were the most frequently identified pathogens, we found a high proportion of cranial SSI caused by gram-negative aerobic bacilli. This has been reported in some other cohorts [31–34], although not all [13, 35]. There may be several reasons for the preponderance in our cohort. First, a significant proportion of patients had empirical preoperative antibiotics on presentation with SSI, which covered only gram-positive organisms. This may have increased the relative proportion of GNB identified. It is also credible that environmental gram-negative organisms may have contributed, as indicated in other studies [36, 37]. It is possible that the proportion of GNB was inflated by the number of cases where an organism was not identified. It is also notable that 62% of patients had ≤2 operative samples sent for microbiology culture, and in 5 cases no samples were taken. Some gram-positive organisms may not have been identified as a result; of note, we identified only 1 case of streptococcal infection and no cases of fungal infection. Whether these or other fastidious organisms could have accounted from some of the 20.2% of cases in our cohort where no pathogen was identified is not clear. Published literature suggests that the fungal infections are rare; however, given the modest size of published cohorts, more epidemiologic data are needed. The number and nature of sampling are not defined in these settings and require further research, particularly in cases of infection caused by fastidious organisms [38]. In our opinion, an approach to sampling similar to that recommended in prosthetic joint infections should be considered [39]. The diverse range of organisms found in infected cases at our center highlights the need for local SSI surveillance for neurosurgical procedures, and units should be aware of their local resistance profile in deciding the optimal empirical antimicrobial agents to reduce the risk of postoperative infection.

This study has several limitations that affect interpretation. Our cohort was retrospective in nature, and as such, the reasons behind specific recommended surgical decisions and antibiotic plans were not always clear. Also, although some of the primary surgery was not performed at our trust, all infection management was completed at a single center, so the results may not be generalizable to other centers. Our approach to case definition was designed to capture only cases with clear macroscopic evidence of infection. There was a limited number of microbiological samples available in some cases; as a result, it is possible that the causative organism was not identified, particularly those organisms that are fastidious. The use of histology was also not routinely performed, so cases of osteomyelitis may have been missed. This highlights the need for an agreed postoperative infection definition, which utilizes multiple data sources and a standardized approach. We are currently conducting a national survey to gain better insight into the range of practice across the United Kingdom. This study took a pragmatic approach to defining adverse outcome and included individuals who had died within a year of their infection surgery. As malignancy was a significant indication for primary surgery, it is likely that mortality in these individuals may be attributable to noninfectious factors. Cases for this study were identified from emergency and elective theater lists, but there remains a risk that some cases may have been missed through misclassification, although we think that this is unlikely to have introduced bias. Some patients completed long-term follow-up in other centers in the United Kingdom, resulting in incomplete long-term neurologic outcome data. We were therefore not able to incorporate neurologic outcome into the definition of adverse outcome. We accept that this is an important aspect of defining a successful treatment regimen and should be explored in future studies.

We have reported the infection outcomes for a cohort of patients requiring return to theater for an SSI, showing a high proportion of treatment failures, and we highlight an association with concomitant corticosteroid therapy and previous radiotherapy. Although the most frequently identified organism was *S aureus*, we also noted a large proportion of GNB infections, which highlights the need for tailoring empirical antimicrobial coverage to local microbiologic patterns in SSI. Identifying patients at greater risk of treatment failure remains challenging.

This work highlights the need for a standardized and reproducible case definition for cranial SSI. A widely accepted definition, with appropriate sensitivity and specificity, would underpin robust surveillance and high-quality clinical studies, generating the evidence needed to determine the effect of early surgical debridement and the optimal antimicrobial regimen, route, and duration on patient outcomes. The development of a national consensus guideline would represent an important step toward standardizing care and supporting a more evidence-based, coordinated approach to the management of this complex SSI.

## Supplementary Material

ofag369_Supplementary_Data

## Supplementary Data

Supplementary materials are available at *Open Forum Infectious Diseases* online. Consisting of data provided by the authors to benefit the reader, the posted materials are not copyedited and are the sole responsibility of the authors, so questions or comments should be addressed to the corresponding author.
