# Supplementary material for: Outcomes of Surgical Site Infection Following Cranial Surgery From a UK Tertiary Center
Source: Open Forum Infect Dis. 2026 Jun 20;13(7):ofag369. doi: 10.1093/ofid/ofag369 (PMC13334327; doi:10.1093/ofid/ofag369)
Supplement: ofag369_Supplementary_Data [file ofag369_supplementary_data.docx]

Supplementary Appendix

| **Organism Group/Organism** | **N = 89** | **Craniectomy OR** | **p** | **Cranioplasty OR** | **p** | **Craniotomy OR** | ***p*** |
| --- | --- | --- | --- | --- | --- | --- | --- |
| Gram Negative Bacilli | 22 (25%) | 4.05 (1.27 – 13.54) | 0.019 | 1.11(1.88 – 29.55) | 0.870 | 0.31 (0.11 – 0.88) | 0.030 |
| Gram Positive Bacilli | 20 (22%) | 0.29 ( 0.04 – 1.17) | 0.122 | - |  | 9.75 (2.46 – 65.50) | 0.004 |
| Anaerobes | 4 (5%) | 1.13 ( 0.05 – 9.61) | 0.916 | - |  | 2.08 (0.25 – 43.21) | 0.536 |
| Coagulase Negative  *Staphylococci* | 15 (17%) | 1.30 (0.32 – 4.63) | 0.692 | 1.00 (0.31 – 3.35) | 1.000 | 0.69 (0.10 – 3.07) | 0.660 |
| *S.aureus* | 21 (24%) | 0.73 (0.28 – 2.45) | 0.620 | 6.92 (1.88 – 29.55) | 0.005 | 0.36 (0.12 – 1.02) | 0.059 |
| *Cutibacterium acnes* | 19 (21%) | 0.31 (0.05 – 1.28) | 0.008 | - |  | 8.84 (2.23 – 59.45) | 0.006 |
| *S.epidermidis* | 10 (11%) | 2.67 (0.60 – 10.93) | 0.174 | 0.49 (0.03 – 3.06) | 0.525 | 0.62 (0.16 – 2.46) | 0.488 |
| *Pseudomonas aeruginosa* | 5 (6%) | 0.83 (0.04 – 6.19) | 0.875 | 3.67 (0.44 – 25.01) | 0.183 | 0.42 (0.05 – 2.68) | 0.356 |
| *Escherichia coli* | 4 (5%) | 3.71 (0.42 – 33.29) | 0.209 | - |  | 0.65 (0.07 – 5.69) | 0.676 |

*Table 1. Associations between cranial SSI type, organism group and the five most commonly isolated organisms. Data presented as Odds Radio (95% Confidence intervals and p value)*

| Antibiotic | | Additional Agent | | Cranioplasty | % | Craniotomy | % | Craniectomy | % |
| --- | --- | --- | --- | --- | --- | --- | --- | --- | --- |
| Ceftazidime | IV | Vancomycin | IV | 10 | 63 | 29 | 53 | 13 | 59 |
| Ceftriaxone | IV |  |  | 0 | 0 | 3 | 5 | 2 | 9 |
| Ceftriaxone | IV | Metronidazole | IV | 0 | 0 | 1 | 2 | 0 | 0 |
| Ceftriaxone | IV | Metronidazole | PO | 0 | 0 | 1 | 2 | 0 | 0 |
| Ceftriaxone | IV | Vancomycin | IV | 0 | 0 | 1 | 2 | 0 | 0 |
| Co-amoxiclav | IV |  |  | 4 | 25 | 14 | 25 | 3 | 14 |
| Co-amoxiclav | IV | Vancomycin | IV | 0 | 0 | 1 | 2 | 0 | 0 |
| Co-amoxiclav | PO |  |  | 2 | 13 | 1 | 2 | 0 | 0 |
| Flucloxacillin | IV |  |  | 1 | 6 | 0 | 0 | 2 | 9 |
| Meropenem | IV |  |  | 0 | 0 | 1 | 2 | 1 | 5 |
| Meropenem | IV | Vancomycin | IV | 0 | 0 | 3 | 5 | 1 | 5 |

*Table 2. Initial post operative antibiotic regimen used separated by site of SSI. Percentages express proportion of site group.*
